# Microbiome Yarns: human biome reproduction, evolution and visual acuity^1^,^2^,^3^,^4^

**DOI:** 10.1111/1751-7915.13037

**Published:** 2017-12-26

**Authors:** Kenneth Timmis, Franziska Jebok, Gabriella Molinari, Manfred Rohde, Leo Lahti

**Affiliations:** ^1^ Institute of Microbiology Technical University Braunschweig Braunschweig Germany; ^2^ Institute for Educational Science University of Freiburg Freiburg Germany; ^3^ Central Facility for Microscopy Helmholtz Centre for Infection Research Braunschweig Germany; ^4^ Department of Mathematics and Statistics University of Turku Turku Finland

## Part 1

Los Angeles, November, 2027.

*BBZ, Studio 7A, GET Plaza, Burbank, 7.30 pm: Abigail Repor‐Tastory* 5 *, Discovery Presenter, turns to face the camera*: Good evening and welcome to a new episode of ‘Microbiome Discoveries that Change our Lives’. Our guest this evening is once again Dr. Anastasia Noitall‐Most^6^^ ^from the Streber Elite University of Los Angeles^5^. Good evening Dr. Noital‐Most *(shaking hands)* and thank you for appearing on the program.

*Dr. Noitall‐Most:* Good evening Abi; it is always a pleasure to be here.

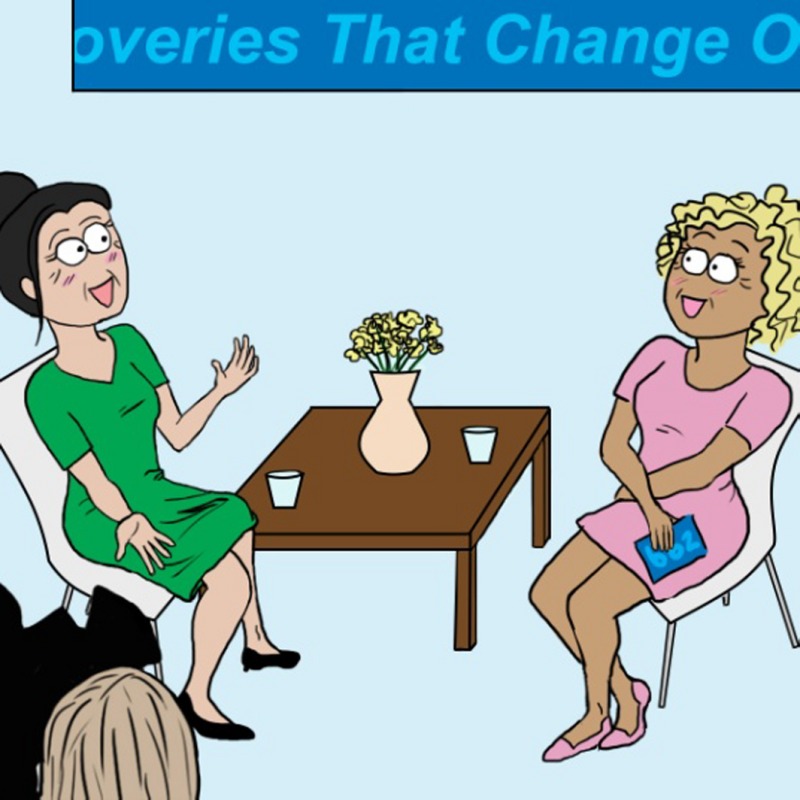

*Ms. Repor‐Tastory*:* *Ani, this evening we will discuss some remarkable developments on different fronts that have recently been connected in a controversial proposal made by a respected member of the microbiome research community.

*Dr. Noitall‐Most:* Yes, Abi: the proposal, which is published in the renowned journal Microbial Biotechnology^7^ is about human evolution. If it turns out to be true, it will change our view of the role of our microbial partners forever.

*Ms. Repor‐Tastory:* Okay, please tell us more.

*Dr. Noitall‐Most: *Gladly, but first some background. As you know, a few years back, the whole world gained access to smartphones. The reason for this was the development of a new business model by smartphone companies: instead of selling phones at high prices only to high income families, they distributed them free to essentially everyone on the planet able to hold a phone the right way up and click a button. In advance of, and in preparation for, implementation of their new business strategy, the cash‐rich, smartphone‐tech developers had bought and assumed operation of major telecoms and pay operators, like Chargemate, and subsequently charged people on an all‐inclusive or pre‐defined package basis. Thus, there was a massive explosion in smartphone use, which continues to this day.

*Ms. Repor‐Tastory:* Yes, Ani, and we can upgrade to the latest model whenever we want, and only have to pay for personalisation, like the latest colours, materials, sensors, apps, etc. My new one has the newest ultrasound option which allows me to check for osteoporosis; expensive but well worth it, given my family history!

*Dr. Noitall‐Most:* Quite so! Moreover, the two leading smart device companies also bought up major health data operators, and analyse the flood of health data collected and transmitted via the smartphones, wearables, and the *internet of things*. At a stroke, they became the major players in health profiling in personalised medicine.

Anyway, as we also know, for more than a decade, biomedical researchers have been carrying out longitudinal analyses of the microbiota of a number of body sites, along with corresponding clinical parameters, to obtain insights into the possible roles of our microbes in body functions and health and disease. Well: one study carried out by Drs. B. L. Eary and O. Culah, both coincidentally named by their mothers after patron saints of vision, St. Lucia^8^ and St. Odile^9^, of the Centre for Ocular Microbiology in Belfast, which investigated possible relationships between visual acuity – how well we see – and the microbiota of the conjunctiva – the outer surface of the eye and the inner surface of the eyelid – and the tear gland which lubricates these surfaces, led to a disturbing finding. Apparently, progressive deterioration of vision, which used to be primarily a problem of our older citizens, is increasingly also affecting younger people in the 20 +  age group.

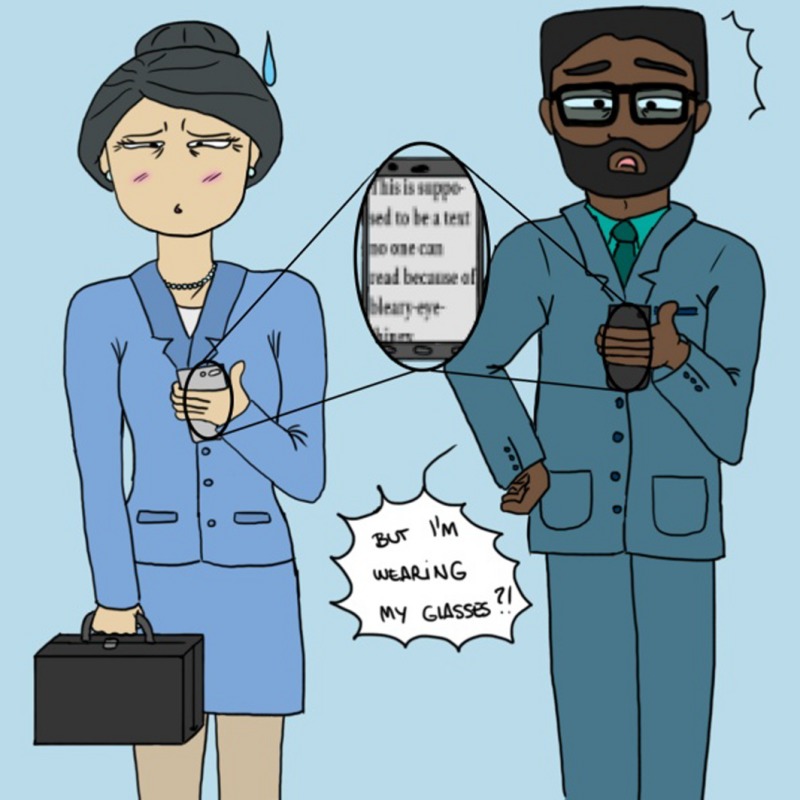

While the worsening trend of acuity deterioration has been observed by ophthalmologists for some time, it was assumed to be caused by eye strain resulting from constant peering at smartphones and tablets while keeping up with social media activities.

*Ms. Repor‐Tastory:* Yes, I know what you mean! I sometimes get quite blurry eyed after an hour or so of scrutinising the new selfies posted by my friends and competitors. And not only because of jealousy.

*Dr. Noitall‐Most: *Absolutely! Another explanation, recently proposed by a famous French scientist, and rapidly gaining ground, is that the trend towards excessive political correctness is increasingly preventing us from seeing things as they are – we are becoming visually impaired through optical distortion^10^. But the new findings relegate both hypotheses to the waste bin.

*Ms. Repor‐Tastory:* Wow! So please tell our viewers what the Belfast group discovered.

*Dr. Noitall‐Most: *Right: so what Lucia and Odile found was a new cause of ocular impairment that is rapidly increasing in frequency. The aetiology of this new malady was investigated in collaboration with the Imaging Group of Mabriella Golinari and Ranfredy Mohde of the Walpur Gisnacht Institute for Cellular Pathology in Bad Hurzbarg in Northern Germany, and revealed to be a new bacterium colonising the cornea which produces copious amounts of a slime that reduces visual acuity by creating a translucent film over the corneal surface. This bug was given the name *Phonescreenia blearyeydi* 11.

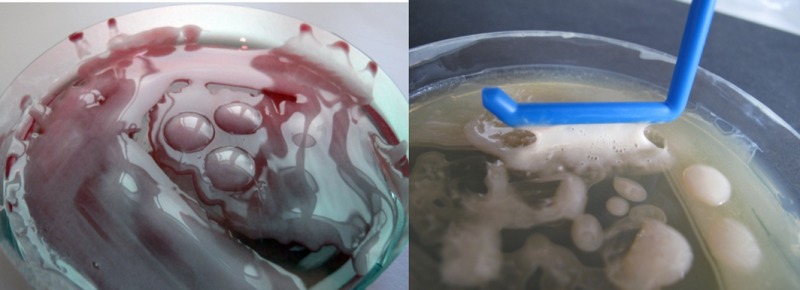

But the thing is this: although *Phonescreenia blearyeydi* is easily treated with antibiotics, young people are refusing to have any antibiotic intervention that is not absolutely essential – i.e. lifesaving – because of the antibiotic resistance scourge. As you know, in 2016, the World Health Organisation sounded the alarm of the pending antibiotic resistance catastrophe that would basically render potentially lethal infections untreatable^12^. For this reason, young people have been accepting poorer vision rather than take the risk of subsequently contracting a life‐threatening infection.

*Ms. Repor‐Tastory:* Golly! But how can the smartphone provoke the growth of *Phone*…whatever it is called?

*Dr. Noitall‐Most: *Well, it seems that the eye does not like to be set at a single focal length for long periods of time. Indeed, it developed evolutionarily for the hunter‐gatherer who was a predator among other predators and who needed to be constantly scanning near and far vistas for opportunities, like food, potential mates and hazards, so the eyes were continually adjusting to changing focal lengths. In the world of the smartphone, however, the eye is trained for very long periods of time on an object always some 25 cm from the retina, so the muscles controlling both eye focussing and movement are not exercised, or at least only within a certain range determined by the smartphone. Of course, a variety of eye exercises have been proposed to compensate for this^13^, but the effectiveness of some of these is controversial and, anyway, most people either forget or do not have time for them. Importantly, what the researchers found was that, in people who spend a lot of time on smartphones, small amounts of a new metabolite are secreted into the tear glands, and thence onto the cornea. It turns out that this metabolite stimulates both the growth of *Phonescreenia blearyeydi,* and its production of slime; they called it iGROWF, for eye growth factor. iGROWF secretion is the physiological response to insufficient eye muscle exercise.

*Ms. Repor‐Tastory:* So what will be the long term consequence of this?

*Dr. Noitall‐Most: *Well, before we get to this, an important, related discovery needs to be brought into the picture. This is that birth rates are going up again, which is amazing, given that fertility had been falling steadily over recent years^14^, ostensibly due to the accumulation of xenoestrogens (estrogen‐like) and other endocrine‐disrupting chemical pollutants in the environment, and the food chain^15^. This unexpected change in trend has been investigated by Dr. Fecunditas – *Ditty* for short – Amoretto of the Genghis Institute of Reproductive Biology in Rome, who found that, contrary to expectation, fertility has been increasing rather dramatically in recent years. Moreover, the endocrinological profiles of younger adults are also changing and are consistent with higher libido levels and sexual activity.

*Ms. Repor‐Tastory, wickedly:* Gosh! Are you saying that there really is a relationship between sexual activity and our ability to see? So the old saying *is* correct!

*Dr. Noitall‐Most, smiling: *No, not really: despite some well‐publicised exceptions^16^, too much sex does not make you blind, although the converse may well be true: poor vision does seem to increase interest in carnal activity.

*Ms. Repor‐Tastory:* Ah – is this a variant of the non‐functioning television story?

*Dr. Noitall‐Most:* Absolutely, Abi! Anyway, the big question is: what is the cause of this new endocrinological trend? Serendipitously, Dr. Amoretto met Joe Lee Swagman of the Australian National Institute of Marsupial and Rodent Psychology in Alice Springs at a symposium on *Microbiome Mediators of Behaviour* in Reno, Nevada. During the meeting, Ditty and Joe became, shall we say, networked, which created an environment for pillow talk that *inter alia* included discussions about current endocrinological and microbiomological developments in young adults. These discussions led them to realise that there was a correlation between microbiota changes recorded recently and the increases in fertility. So they decided to investigate together the possibility that this correlation reflects a causality. The first thing they did was to use an ultra‐sensitive metabolome profiling system to look for metabolically‐active small molecules in the intestinal tract of young adults, and to compare these with corresponding levels of libido, frequency of sexual activity, and frequency of pregnancy. Interestingly, they found a new testosterone‐like compound, or TLC, in many subjects, the levels of which correlated nicely with levels of libido and frequency of sexual activity, in both male and female subjects, and with pregnancies in females.

They then teamed up with the Irish and German groups to form an international research consortium which used high throughput single cell cultivation and metabolite screening to identify the microbes producing TLC. This resulted in the isolation of a bacterium that was easy to cultivate in the laboratory and produced large amounts of TLC. It turns out that this bug is present in almost all people, mostly in low numbers, but in higher numbers in young people with high libido. With a specific labelled antibody, it was possible to show that this bacterium is almost always found in small microcolonies on the surface of the epithelial cells of the small intestine – the so‐called brush border of the enterocytes – which would favour maximal entry of the hormone into the intestinal blood capillary system. Dr. Amoretto named this bacterium *Casanovia resplendenti* 17, or CNR for short.

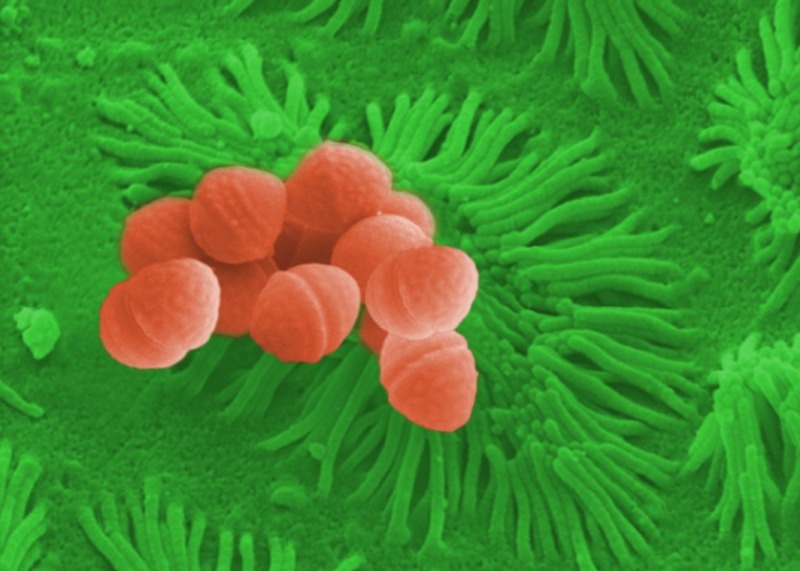

The intrepid researchers isolated pure TLC in quantity from CNR, determined its chemical structure, and Joe Lee Swagman then investigated its activity by introducing it into the intestines of laboratory mice.

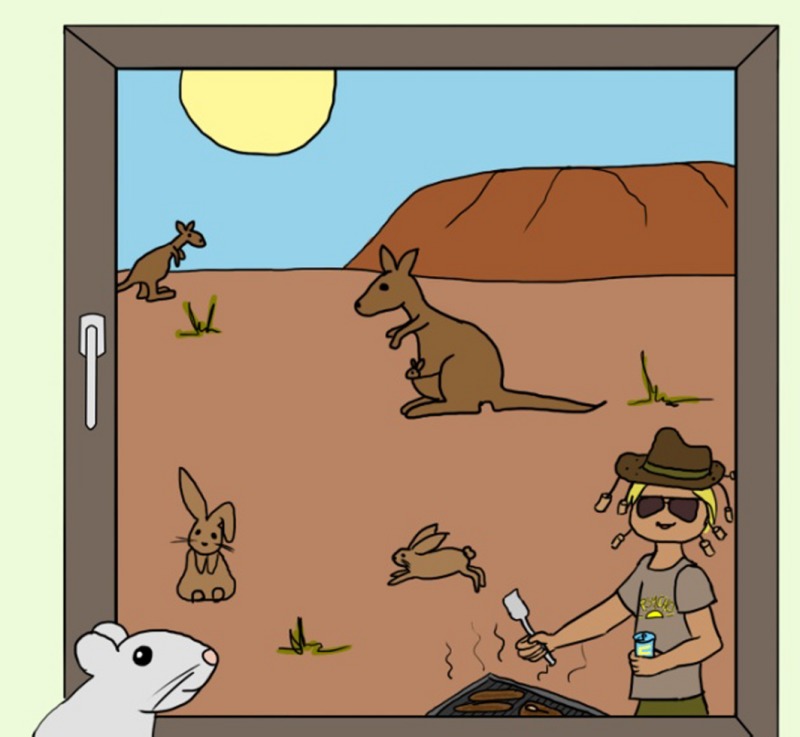

Since Joe is a good multi‐tasker, he was able to monitor mouse behaviour in the experiment while simultaneously organising a barbie for his lab mates and consuming several beers. The result of the experiment was that, whereas control mice behaved normally, those receiving TLC went into a frenzy of copulation that did not stop until they were either exhausted or grimacing with pain.

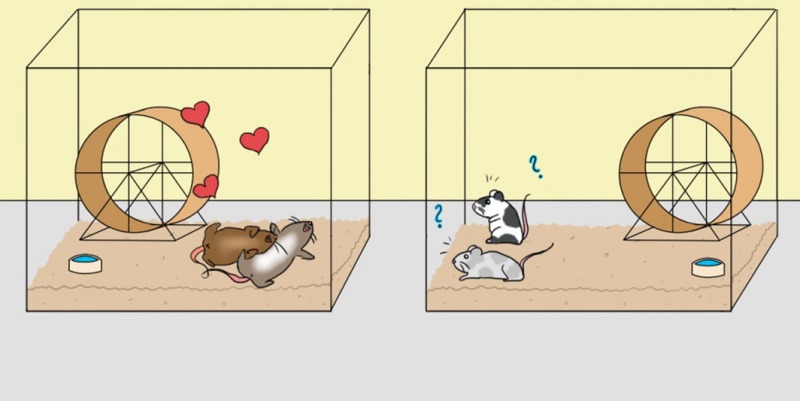

This series of experiments suggested that TLC is a powerful libido stimulant and that the increased libido seen in young people is most likely due to higher levels of CNR in their intestinal tracts.

*Ms. Repor‐Tastory, facial expressions signalling conflicting emotions:* Gosh! Er…will TLC become available to the general public at some point?

*Dr. Noitall‐Most, squinting at Abi: *Yes, Abi, many people are asking this question and, of course, several companies are actively exploring the commercial opportunities available, both for the natural product and for synthetic derivatives.

But to get back to the story. Since the results were obtained in an animal model under controlled laboratory conditions, the team wanted to assess the situation in humans in their natural settings. They therefore carried out an analysis of TLC levels in faecal samples of a large cohort of volunteers, who also provided detailed information on their activities that were queried in an extensive questionnaire. As you know, in societies where there are no restrictions on the choice of partners, the Pareto Principle^18^ states that 20% of young heterosexual men will bring to bed 80% of available heterosexual women, and 20% of young heterosexual women will bring to bed 80% of available men.

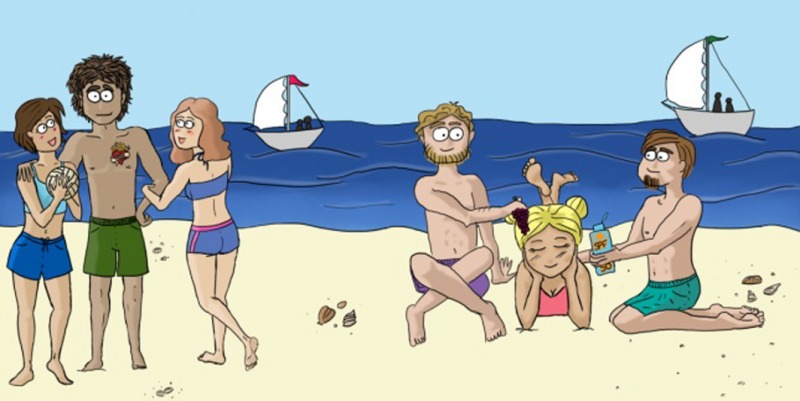

So, in the analysis of the questionnaires completed by the study cohort, the 20% men and 20% women who were particularly sexually active were readily identified and compared with the other group of subjects characterised by somewhat more restrained behaviour. They then enlisted the aid of the renowned mathematician, Professor Fidget Jones^19^, in the analysis of the results. As a result, he was able to derive a term, the Frequency and Diversity Index of Procreation Activity, or FDIPA, that categorised the subjects^20^.

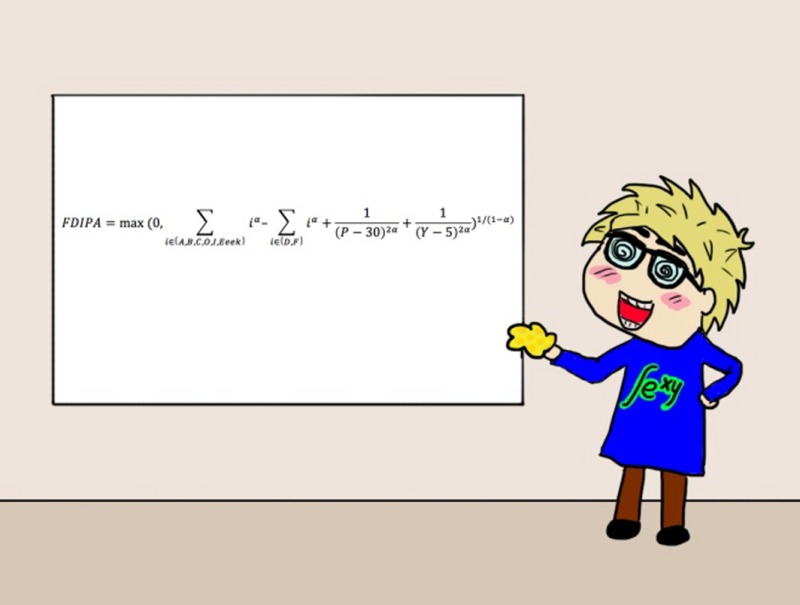

The conclusion of this study was convincing: the Pareto 20% groups, characterised by high FDIPA values, contained up to 100x more TLC‐producers than the 80% ‘control group’, characterised by average FDIPA values.

*Ms. Repor‐Tastory, a little too enthusiastically:* Gosh! Do the 20%ers look any different from the 80%ers?

*Dr. Noitall‐Most: *Well, Abi, I have no idea. I do not believe that documentation of physical attributes was part of the study design. But future studies will surely include additional, relevant parameters like these.

Anyway: to continue. The team then examined some historical faecal sample collections that had been conserved well at −80°C or lower, in order to see whether the average TLC content has changed over time. And indeed, what they found was that TLC levels had been rather constant over several decades prior to, and one decade into, the mass acquisition of smartphones and other devices, and the explosion in social media, but then sharply increased over time for about a decade, more or less in parallel with the increase in problems of visual acuity in young people.

*Ms. Repor‐Tastory:* Well, on that note, we'll take a break and return in a few minutes to this fascinating story to learn more about the role of our microbiome in vision, reproduction and evolution of the human biome.

## Part 2

*Ms. Repor‐Tastory: *Welcome back viewers! Okay, Ani: you have given us a lot of information, but what does it all mean?

*Dr. Noitall‐Most: *Well, Abi: at this point, we cannot be absolutely certain, but there is an interesting theory, advanced by Professor Tim Kennis of the Queenton Institute of Advanced Studies and published in Microbial Biotechnology^7^, which is rapidly gaining acceptance. His starting point is the fact that the primordial, quintessential property of all living things is reproduction – an organism that does not reproduce sufficiently rapidly goes extinct. Essentially all other features of living organisms serve to mediate growth and reproduction. The only exception is the human, who additionally allows himself the privilege of a few less‐ or non‐reproduction‐contributing activities, like stamp collecting, writing computer programs, knitting, doing tax returns, mowing the lawn, watching quiz shows, playing bingo, tinkering with old cars, etc., but even then these are always peripheral to the main business of growing (= eating) and reproducing.

So: while it is true that the global community continues to grow at an alarming rate, given the limits of available of resources^21^, some populations, like those of Southern Europeans, have for a long time been experiencing falling birth rates and thus were on a trajectory towards extinction^14^. Of course. evolution will tend to react to this and adaptively compensate in some way. However, evolution is generally considered to be a slow process, with observable change needing many generations. For humans with reproduction rates of 20 years, give or take, this is hundreds of years. However, Kennis suggests that, although we have always previously considered human evolution to involve only the human being itself, in reality the entity upon which natural selection works is the human biome. Unlike humans, members of the human *micro*biome reproduce in minutes‐hours so, in fact, microbiome‐influenced characteristics can evolve rapidly.

Thus: according to the scenario developed by Professor Kennis, the human microbiome has somehow recognised that its reproduction rate is lower than sustainability requires, placing it on a trajectory to extinction, and has therefore taken robust steps to correct this, to increase reproduction rates, and thereby accelerate the process of human evolution.

Because of the spectacular increase in smartphone usage, connectivity and social media, people spend a major part of their lives looking into the smartphone/tablet, which is time they previously used for other things, including procreation. Our *carnal footprint* became smaller. It is often said that sex is on the wane because of increasing stress in our lives which reduces libido. However, reduced opportunity in time and place is also a significant contributing factor! Previously, we were much more spontaneous and opportunistic, and used to consecrate considerable effort and resources in the plotting of *when* and *where*: the logistics of coupling constituted a central preoccupation of our lives. But the loss of time that became dedicated to activity on social media practically eliminated both spontaneity and logistical orchestration, thereby reducing carnal activities to quick fixes for all but dedicated fundamentalists.

Well, Professor Kennis concludes that this did not sit at all well with our microbial friends who became increasingly frustrated watching the – mostly inane – unproductive social traffic on the smartphone screen, while all the time willing us to get on with the business of fulfilling our contractual biological obligations. Or, in the context of a current theory we discussed in an earlier program^22^, namely that humans may simply be microbially‐designed and controlled scaffolds for microbial habitats, the microbes will not permit non‐reproductive lifestyles! So: as soon as the aberration looked as though it would continue for a while, our friends plotted mitigation strategies and orchestrated our rapid evolution. This is classical Gaia – the normal biological compensatory response to functional change^23^.

Kennis proposes that our microbiome friends organised themselves into a sort of microbial action group against smartphones, and has coined the term MAGASP for this. He suggests that MAGASP developed three strategies to compensate for inadequate birth rate levels, namely changes that increase intimacy, libido, and fertility.

The first is an increase in the deterioration of visual function in people of reproductive age, which previously had been a health issue mostly in people well past the age of reproduction. The consequence is, of course, that folk find it less enjoyable to look at their phones for extended periods, gain more free time and, at least subliminally, consider other activity options to fill the gap, including fun between the sheets.

The second, as we have seen, is the general increase in population sizes of CNR‐type intestinal bacteria that produce TLC which mediates increased libido.

*Ms. Repor‐Tastory, with a wicked expression:* Yes, Ani: but surely an increased libido does not in of itself translate to increased fertility? In my personal experience, Southern Europeans have always enjoyed a more than respectable libido, despite the fact that their birth rates were falling. During a recent holiday in Sorrento, I met this incredibly ….*……(angry noises emanating from the in‐ear headphone)*…Oh, sorry, I did not mean to interrupt…

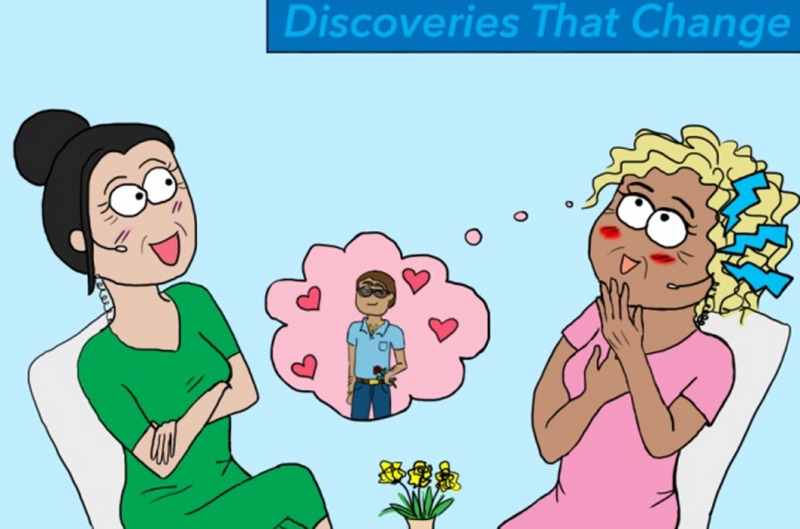

*Dr. Noitall‐Most: *Of course, Abi, you are absolutely correct. As I mentioned earlier, falling fertility has been assumed to be the consequence of the accumulation of xenoestrogens (estrogen‐like) and other endocrine‐disrupting chemical pollutants derived, for example, from plasticisers used in the production of petrochemical‐based bulk polymers, in the environment, from which they enter the food chain^15^. Because of the highly serious consequences of endocrine‐disrupters, there has been a lot of research to find microbes able to degrade such compounds^24^. While degraders have been found, they have not until recently been shown to be useful, because the concentrations of these hormone‐like pollutants in the environment are very low, despite being highly active. Low concentration pollutants have traditionally been ignored by our microbial friends who, like us, prefer to eat more accessible tasty items of food. Now, however, Professor Polly C. H. Lorinate, Chief of the Microbiology Detoxification Group at the Environmental Chemical Research Center in Leipzig, has shown that a bacterial degrader of endocrine disrupters, which they named *Oestrogobbla furiosus* 25, has established itself in the human gut flora and completely degrades these compounds even when present at very low concentrations. So: the third string in the microbiome bow is bacterial removal of agents that depress fertility; as a result, the fertility of young people is increasing.

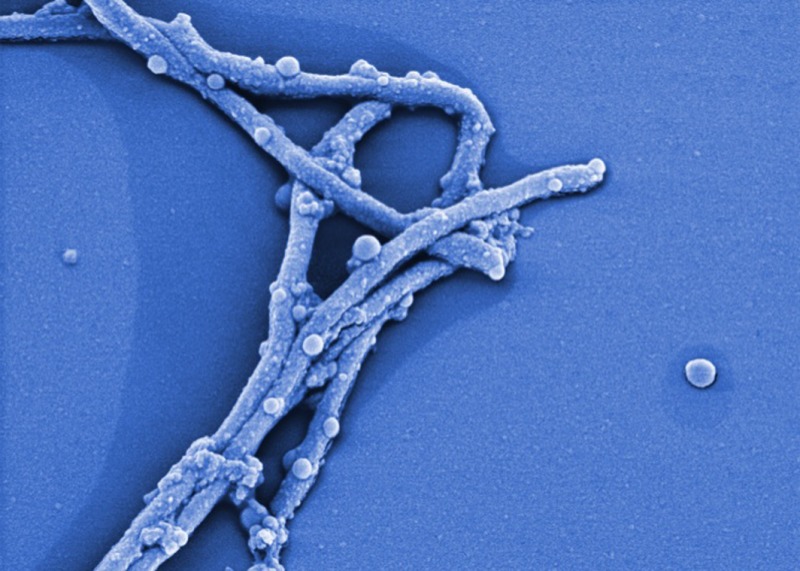

*Ms. Repor‐Tastory:* Well, Ani: rapid evolution of humanity via its microbiome is indeed an exciting, if slightly worrying, prospect. Are we all condemned to poor eyesight?

*Dr. Noitall‐Most: *Of course, that is the central question, and cannot yet be definitely answered. However, follow‐up studies seem to suggest that, as birth rates go up, so the frequency and severity of impairment of visual acuity go down. Thus, it seems that our microbial friends recognise the basic evolutionary utility of smartphones and will (a) allow their reduced use, without compromising our ability to see the Mont Blanc when there is no cloud cover, etc., but (b) retain the capacity to punish us for, and correct, any habit we develop that compromise in any way their (and our) reproduction rates.

*Ms. Repor‐Tastory:* Well, this is all rather sobering! Kennis’ notion that our microbiome orchestrates our evolution seems rather radical: is his proposal generally accepted by experts in the field?

*Dr. Noitall‐Most: *Yes, Abi, it is becoming widely accepted, not least because there are many examples in biology of microbes known to influence animal and plant behaviour. For example, the symbiotic bacterium *Wolbachia* can even determine the gender of its insect host^26^.

*Ms. Repor‐Tastory (with a look of horror and shifting uncomfortably in her chair):* Ooohh: how creepy! Are there any other consequences of these findings?

*Dr. Noitall‐Most: *Yes, Abi, there are. Indeed, a most interesting aspect of these discoveries is an unexpected plethora of knock‐on effects that greatly exceeds those of other microbiome discoveries we have so far reported on this program.

The first is that now everybody and his dog know about microbiomes and their dominant role in our physiology and well‐being. This heightened awareness is obviously a good thing and contributes generally to healthcare literacy in the general population which, in turn, encourages adoption of healthy lifestyles^27^. For example, only the other day, a leading microbiome researcher, Professor Gill Jackbert, told me that he has many requests from schoolchildren to undertake vacation internships in his group to determine their microbiomes and biome‐health profiles.

Also, the reduced usage of smartphones has not only led to an increased appreciation of the visual pleasures of our environment, but concomitantly also of the other senses. So, for example, we see far more folk out and about without music streaming into their ears, appreciating the sounds of the country, the joyful squeals of children in the playground, the dawn and evening bird chorus, etc. And there has been a substantial reduction in road accidents, especially those of cyclists, as a result of being able to hear approaching traffic. There is, however, one small hard core group who persist in constant in‐ear bombardment of the senses, namely teenagers, presumably to avoid hearing their parents reminding them to get up for school, finish their homework, etc. However, this is just a phase and they grow out of it.

*Ms. Repor‐Tastory: *Of course, Ani….. I vividly remember that, as a teenager, I had an absolute need for constant music for my well‐being.

*Dr. Noitall‐Most: *Moreover, the change from the smartphone being our primary environment, to the environment being our environment, has had a few other consequences. One is that partnerships which originated and prospered through the smartphone – we all know couples who met on the internet, spent all free time on the phone, including quality time like a dinner date in a favourite restaurant, or on breaks at scenic spots on a hike, and frequently communicated with one another via social media – suddenly became objectively appraised though a full complement of senses, in the cold light of day so to speak, and were found, often accompanied by considerable surprise, to be wanting. There has thus been a flurry of separations of couples and re‐assortment of relationships, based on old fashioned parameters, like mutual attraction, common (non‐phone) interests, material assets, new citizenship opportunities, and so forth.

*Ms. Repor‐Tastory:* Well, there are certainly a multitude of consequences of less phone usage. However, before we bring our discussion to an end, I should like to pose my usual final question: are there economic consequences of this work?

*Dr. Noitall‐Most: *Indeed there are, Abi!

As we have seen, the changes in the business models of smart tech companies, and a massive expansion in ownership of phones and other smart connected devices, and their use, directly induced a Gaia response that involved our microbiome inducing impairment of visual acuity and, as a consequence, reduced phone usage and hence profits. Well, the new phone‐smart tech companies, which have added telecom operations, e‐money collection, and personal data analysis services to their portfolios, are some of the most innovative and nimble business folk around. Their response to falling phone usage has been to increase involvement in health data collection, analysis and marketing of personalised medical profiles and healthcare recommendations. Moreover, two major players each bought up a leading pharma company and, more importantly, numerous start‐ups, and are beginning to develop and market new pharma products based on their own analyses and recommendations. These erstwhile smartphone/tech developer companies are now predicted by some to become the major pharma industry players of the future. And, of course, the first products they are developing are ophthalmological products to prevent or treat deterioration of visual acuity. They say they do this because of their deep sense of corporate responsibility to solve problems they created, but more cynical types observe that creating a market to satisfy with a new product is standard business practice and that, anyway, improvement in visual acuity will increase phone usage.

Of course, a major interest is the economic potential of TLC and its derivatives. Interestingly, one major player in the commercial development of TLC‐based products is the company PULPS^28^, which markets exclusive products developed primarily for the more senior members of well‐to‐do society. Clinical trials thus far carried out on both females and males are highly promising. Not only are low doses of TLC extremely effective at errr *(coughing discretely)*, let's say, encouraging folks to indulge, but also seem to reduce smartphone dependency. The test cohort, in contrast to the control group, seemed more interested in their partners than in their virtual social network, which is truly a reversal of the previous trend. Already, with the naturally‐elevated levels of TLC among young people, our *carnal footprint* has increased, accompanied by parents walking their prams or playing with infants in the park, without a sight of a phone and showing obvious pleasure and pride in parentage and interactions with the little things. This behavioural change contrasts with the previous situation, when most parents existed in a parallel world of their smart devices and ignored their children, and also with the behaviour of the control group. So: family life as it once was, is starting to reappear, and will definitely increase when TLC comes on the market.

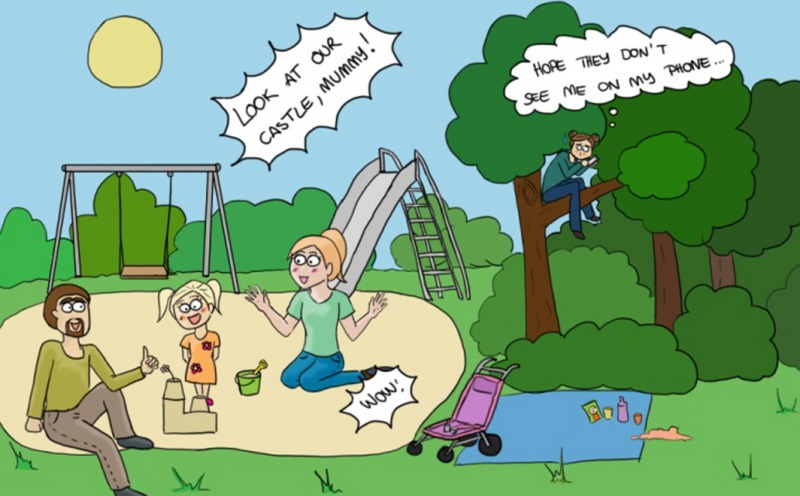

*Ms. Repor‐Tastory: *Well that is just wonderful! So there is a fairy tale ending to the problem of poor eyesight!

*Dr. Noitall‐Most: *Indeed, Abi. In this vein, I might also mention that Ditty and Joe Lee are now happily married and have three children! And in a similar vein, another application, though not a microbiological one, is the business of romance. A major consequence of the smartphone ‘flood’ was the disappearance of romance, flirting, etc.: there was simply no time and anything outside of peering at the screen was something to be rushed. Now, our microbiome friends have ‘created’ time and inclination. The problem is that we have forgotten about romance and what it entails. So now there is a rapidly growing business, dominated by folk old enough to remember, built around teaching young folk about flirting, romance, love, and physical‐mental‐emotional intimacies (e.g. flowers not just for decoration, dinner in a restaurant not just for business deals, handwritten cards instead of e‐mails, etc.).

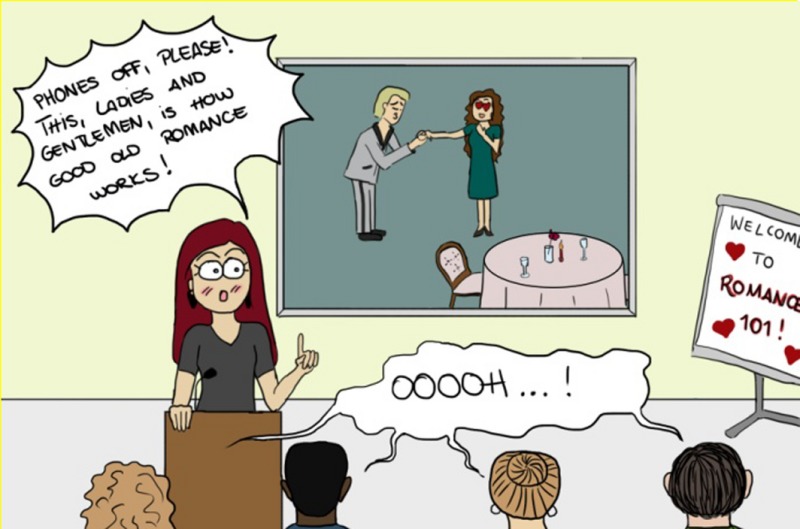

Fortunately, humans are characterised by an exceptional ability to rapidly learn new things, so these almost extinct characteristics will be re‐discovered in a relatively short period of time.

*Ms. Repor‐Tastory: *Yes: the art of romance is the big new trend, but I also hear that there is an explosion in office romances, which is causing a few problems in the workplace.

*Dr. Noitall‐Most: *Indeed, Abi – every silver lining has a cloud.

And, as you are aware, the general movement away from phone dependency has created another business opportunity: phone addiction treatment centres are opening up on practically every street corner.

Another, more global, consequence of these developments is that the overall lowering of visual acuity not only reduced the ability to focus on smart device screens but also the ability to drive, so people are driving less and buying fewer cars. Roads are less congested, so road transportation is more efficient; there is less road wear and tear, so less need for road repairs, construction of new roads, and transportation infrastructure. As a result, there is more tax revenue available for investment in healthcare and the biobusiness‐based economy, especially microbial biotechnology. There is also, of course, less car pollution, less greenhouse gas production and hence a slowing of global warming.

*Ms. Repor‐Tastory: *My goodness – who would have guessed that smartphones would contribute to lower greenhouse gas production (yes, I know: it is not the phones *per se*, but our microbial friends mitigating our destructive behaviour)!

*Dr. Noitall‐Most: *Amen! And last but not least: the slime produced by *Phonescreenia blearyeydi* has now been shown to represent a super new recyclable biomaterial for making glass opaque, for windows and glass doors where a discrete level of privacy is desired. As you know, our economy is becoming increasingly circular and the recycling of materials, especially those used in the building trade, is paramount^29^, and this biodegradable slime is apparently the best thing for ‘opaquing’ since sliced bread

*Ms. Repor‐Tastory: *Well, Abi, such a diverse range of consequences of this microbial Gaia response to smartphones! Thank you so much for revealing how rapidly we humans can evolve if our microbiomes get involved, especially if the evolution concerns our reproduction, and we look forward to having you again as guest on the program in future.

## Conflict of Interest

None declared.

++++++++

## Explanatory Notes for Online Version at www.theabsurdmicrobe.com

5 http://www.theabsurdmicrobe.com/discoveries/

6 youngest daughter of the renowned Dr. Noitall, see e.g. Koshland, D. E., Jr. 1994. Editorial: A simple proposal for health care rationing. *Science* **264**: 1515

7 Timmis, K., Timmis, J. K. and Timmis, J. K. 2027. Microbiome‐orchestrated and accelerated human evolution: the example of MAGASP. Microb. Biotech. **104**: 69‐96.

8 Santa Lucia: patron saint of vision: http://www.stlucy-campbell.org/about-staint-lucy/

9 Sainte Odile: https://en.wikipedia.org/wiki/Odile_of_Alsace

10 Antoine Danchin, personal communication

11 No, sorry: this is not *Phonescreenia blearyeydi*, but L7, a *Pseudomonas jensenii* strain isolated from plant litter by K. Timmis and characterized by G. Molinari and M. Rohde. Its well‐endowed slime production predestined it to play the role of *P. blearyeydi*, which is of course a figment of our imagination.

12 World Health Organization. 2015. Global action plan on antimicrobial resistance.

13 e.g. http://www.wikihow.com/Exercise-Your-Eyes

14
http://ec.europa.eu/eurostat/statistics-explained/index.php/Fertility_statistics; https://www.irishtimes.com/news/science/why-is-europe-losing-the-will-to-breed-1.2644169

15 e.g. https://www.niehs.nih.gov/health/topics/agents/endocrine/index.cfm; Snyder, S. A., et al. 2003. Pharmaceuticals, personal care products, and endocrine disruptors in water: implications for the water industry. *Environm. Engin. Sci*. **20**: 449‐469; Toppari, J., et al. 1996. Male reproductive health and environmental xenoestrogens. *Environ. Health Perspect*. **104**: 741‐803; Götz, F., Thieme, S. and Dörner, G. 2001. Female infertility – effect of perinatal xenoestrogen exposure on reproductive functions in animals and humans. *Folia Histochem. Cytobiol*. **39**: 40‐43; Possemiers, S. and Verstraete, W. 2009. Oestrogenicity of prenylflavonoids from hops: activation of pro‐oestrogens by intestinal bacteria. *Environ Microbiol Rep*. **1**: 100‐9.

16 Friberg TR, Braunstein RA, Bressler NM. 1995. Sudden Visual Loss Associated with Sexual Activity. *Arch Ophthalmol*. **113**:738–742.

17 No, sorry: this is not *Casanovia resplendenti*, but *Enterococcus faecium* strain E315, isolated by Gabriella Molinari while in Italy and reported in Romero‐Tabarez, M., et al. 2006. 7‐*O*‐malonyl macrolactin A, a new macrolactin antibiotic from *Bacillus subtilis* active against methicillin‐resistant *Staphylococcus aureus*, vancomycin‐resistant enterococci, and a small colony variant of *Burkholderia cepacia*. *Antimicrob. Agents. Chemother*. **50**: 1701‐1709. Its enteric habitat and stunning beauty preordained it for the role of *C. resplendenti*, which is of course a figment of our imagination.

18 Pareto, V. 1896/7. Cours d’économie politique, Université de Lausanne, (https://www.institutcoppet.org/2012/05/08/cours-deconomie-politique-1896-de-vilfredo-pareto); https://en.wikipedia.org/wiki/Pareto_principle, with abject apologies to Vilfredo for this wanton distortion of his Principle. For a microbial example of the Principle, see Pelz, O., et al. 1999. Towards elucidation of microbial community metabolic pathways: unravelling the network of carbon sharing in a pollutant‐degrading bacterial consortium by immunocapture and isotopic ratio mass spectrometry. *Environm. Microbiol*., **1**: 167‐174.

19
http://www.theabsurdmicrobe.com/recurring-characters/

20 terms derived by Fidget from completed questionnaires to develop his, by now, famous formula for FDIPA are:
**Y = age** of sexually active individual
**C = charm** term which encapsulates attractiveness to potential partners; approximated by the number of phone numbers of potential partners in the contacts list
**O = **a term that accounts for regular **opportunities** relating to the degree of exposure to potential carnal partners in the workplace, charity work, social activities (clubbing, partying), hobbies, professional or personal travel (especially conventions); approximated by the number of people in the activity in which most time is spent.
**I = **a term that accounts for **irregular opportunities**, such as student Spring breaks, hen/stag parties, political conventions, to meet potential carnal partners; approximated by the number of selfies taken that are not shared with family
**Eeek = lust factor** that takes into account the degree of chemical‐biological attraction between potential partners and includes consumption of performance‐enhancing drugs; approximated by the number of SMS messages per day to the partner of the day
**B = boldness** and inclination to take risks; approximated by the number of traffic violations per year, averaged over 3 years
**F = Fornication uncertainty** constant – a variable term that takes into account headaches, stress, distractions like smartphone alerts, changes in the weather, preoccupation about the next meal or dinner party, worries about weight gain or the development of crow's feet. It has a fixed value of π^2/3^

**P = Pickle constant** – a variable that compensates for the influence of alcohol intake and that accounts for the opposing effects of recreational drinking, namely that modest amounts lower inhibitions and increase social animation, and hence favour uninhibited encounters, whereas less modest amounts progressively reduce the capacity to competently perform any act requiring more than squinting at the world in amazement. The extreme form of this, prior to permanent shutdown of all essential organs, is ‘pickled’, in which any act of will is impossible. Values range from 6 (least inhibition with maximal coherence) to minus 10 (pickled) and approximated from the average number of alcohol units consumed as follows: 0 units = value of 2, 1‐3 units = 4, 4‐6 units = 6, 6‐12 units = 2, 13‐20 units = 0, and 20+  units = −10.
**D = a demotivation** term relating to professional, personal and social disappointments; approximated by the number of dating refusals per year plus the number of refusals of a salary increase over the previous 5‐year period.
**A = assertiveness**‐forwardness, approximated by the number of notches (male) or discrete marker hearts (female) on the bedpost:
*For those readers whose visual acuity is insufficient to provide a clear view of Fidget’s famous equation on his white board, we reproduce it here in large print and high def.:*

$$\begin{array}{c} \mathrm{FDIPA} \end{array}=max(0,\sum_{i\in \{A,B,C,O,I,Eeek\}}i^{\alpha }-\sum_{i\in \{D,F\}}i^{\alpha }+\frac{1}{{\left(P-30\right)}^{2\alpha }}+\frac{1}{{\left(Y-5\right)}^{2\alpha }}){)}^{1/(1-\alpha )}$$

*in order that its eloquent simplicity may be fully appreciated*. The Pickle factor *P* and Age *Y* have optimal values at 5 and 30 respectively. Deviations from this are associated with decreasing scores. Hence, these variables were centred at the optimal value, squared and inverted. The reduction in performance is approximately linear with *F* and *D*, hence these variables are applied with a negative sign. The cumbersome variable normalization issues are ignored to achieve simplicity and computational efficiency, and only non‐negative scores are allowed before exponentiation. The index is calculated per study subject. This modified diversity equation then provides the frequency of nonzero scores, and overall diversity of the scores with different choices of the *α* parameter; with *α* = 0 we get the frequency of nonzero scores for each subject. Positive values *α* > 0 yield diversity scores with increasing emphasis on the most dominantly scoring questions. The frequency and diversity scores can be compared to sexual activity and the TLC level in faecal samples (*x*), with sex (*s*) as a confounding effect in appropriate generalized linear models.

21 e.g. Timmis, K., de Lorenzo, V., Verstraete, W., Ramos, J. L., Danchin, A., Brüssow, H., Singh, B. K. and Timmis, J. K. 2017. The contribution of microbial biotechnology to economic growth and employment creation. *Microb. Biotech*. **10**: 1137‐1144.

22 Timmis, K., Jebok, F., Molinari, G., Rohde, M. and Timmis, J. K. 2017. Microbiome yarns: microbiome basis of memory. *Microb. Biotech*. **10**: 1283‐1292.

23 Lovelock, J. 2000. Gaia – a new look at life on Earth. Oxford University Press

24 e.g. Yoshimoto, T. et al. (2004) Degradation of estrogens by *Rhodococcus zopfii* and *Rhodococcus equi* isolates from activated sludge in wastewater treatment plants. *Appl. Environ. Microbiol*. **70**: 5283–5289; Haiyan, R., Shulan, J., Ahmad, N., Dao, W. and Chengwu, C. 2007. Degradation characteristics and metabolic pathway of 17alpha‐ethinylestradiol by *Sphingobacterium* sp. JCR5. *Chemosphere* **66**: 340‐346

25 No, sorry: this is not *Oestrogobbla furiosus* but goes under the moniker of 77H. It was isolated by Ken Timmis, Joan Timmis and Balbina Nogales from the phyllosphere of some gorgeous plant or other on the beautiful campus of the University of the Balearic Isles, while KT was a short‐term postdoc of BN, to whom he expresses infinite gratitude, and to the UIB for a grant supporting his stay in Palma. Back in Braunschweig, Gabriella Molinari and Manfred Rohde revealed the fascinating anatomy of 77H and discovered that it is related to *Cellulophaga lytica* (Pati, A., et al. 2011. *Standards Genome Sci*. **4**: 221‐232), which is, however, distinctly less winsome. Its tendency to display an intimate relationship with objects that, in the parallel world of the *Yarns*, might be imagined to be pollutant particles in the process of digestion, made it a perfect proxy for *Oestrogobbla furiosus*.

26 Bhattacharya, T., and Newton, I.L.G. (2017) Mi Casa es Su Casa: how an intracellular symbiont manipulates host biology. *Environ Microbiol* [Epub ahead of print] doi: 10.1111/1462‐2920.13964.

27 e.g. Timmis, J. K. and Timmis, K. 2017. The DIY digital medical centre. *Microb. Biotech*. **10**: 1084‐1093

28 PULPS: Patrician Unlimited Lifestyle Products and Services, a holding company registered in Monaco

29 e.g. see http://onlinelibrary.wiley.com/doi/10.1111/mbt2.2017.10.issue-6/issuetoc

